# Characterization of Monoclonal Antibody–Protein Antigen Complexes Using Small-Angle Scattering and Molecular Modeling

**DOI:** 10.3390/antib6040025

**Published:** 2017-12-15

**Authors:** Maria Monica Castellanos, James A. Snyder, Melody Lee, Srinivas Chakravarthy, Nicholas J. Clark, Arnold McAuley, Joseph E. Curtis

**Affiliations:** 1 NIST Center for Neutron Research, National Institute of Standards and Technology, 100 Bureau Drive, Mail Stop 6102, Gaithersburg, MD 20899, USA; castellanosm@ibbr.umd.edu (M.M.C.); j.a.snyderjr@att.net (J.A.S.); melodylee299@gmail.com (M.L.); 2 Institute for Bioscience and Biotechnology Research, 9600 Gudelsky Drive, Rockville, MD 20850, USA; 3 Biophysics Collaborative Access Team-Sector 18ID, Illinois Institute of Technology, Advanced Photon Source, Argonne National Laboratory, Lemont, IL 60439, USA; schakrav11@gmail.com; 4 Department of Drug Product Development, Amgen Incorporated, One Amgen Center Drive, Thousand Oaks, CA 91230, USA; nclark01@amgen.com (N.J.C.); arnoldm@amgen.com (A.M.)

**Keywords:** antibody, antigen, small-angle scattering, docking, modeling, simulation, Monte Carlo

## Abstract

The determination of monoclonal antibody interactions with protein antigens in solution can lead to important insights guiding physical characterization and molecular engineering of therapeutic targets. We used small-angle scattering (SAS) combined with size-exclusion multi-angle light scattering high-performance liquid chromatography to obtain monodisperse samples with defined stoichiometry to study an anti-streptavidin monoclonal antibody interacting with tetrameric streptavidin. Ensembles of structures with both monodentate and bidentate antibody–antigen complexes were generated using molecular docking protocols and molecular simulations. By comparing theoretical SAS profiles to the experimental data it was determined that the primary component(s) were compact monodentate and/or bidentate complexes. SAS profiles of extended monodentate complexes were not consistent with the experimental data. These results highlight the capability for determining the shape of monoclonal antibody–antigen complexes in solution using SAS data and physics-based molecular modeling.

## 1. Introduction

Understanding protein–protein interactions is a primary goal of structural biology, which can have direct impact on the manufacturing and therapeutic applications of monoclonal antibodies. Antibody–antigen interactions are specific and often have favorable equilibrium properties. With two potential antigen binding sites per molecule, antibodies can provide insights into protein interactions that are not possible with typical proteins with a single defined binding site per molecule. For decades, the majority of experimental data elucidating antibody–antigen interactions has come from studying such complexes in crystals using X-ray diffraction [1,2] and in solution using nuclear magnetic resonance spectroscopy [3,4]. Knowledge of the specific atomic interactions that define these interactions can be used to engineer new antibody molecules to elucidate the nature of the association to add value to improved candidate antibodies in terms of their efficacy and their ability to be manufactured, stored, and administered. In addition, knowledge of antibody–antigen structures in the case where the antigen has the capability to bind multiple copies of the same monoclonal antibody could have important biochemical impacts on the basic biology of the immune response and in-vivo response in therapeutic settings.

Small-angle scattering (SAS) using X-rays (SAXS) or neutrons (SANS) is a valuable method to obtain low-resolution shape information from a large variety of soft-matter systems including proteins. While the number of unique constraints in SAS data is limited, the use of atomistic models to interpret SAS data can offer useful insight as the atomic interactions and molecular topology are valid, physics-based constraints on the models. SAS is often used to provide shape information for problems in structural biology [5,6,7]. With selective or random deuteration of hydrogens one can use SANS via contrast matching to elucidate the shape of independent elements of a multidomain complex [8,9,10].

The sample requirements to measure the SAS of proteins are generally similar to other biophysical characterization methods, with concentrations of 0.5 g/L and higher for measurements in solution [7]. SANS can also be used to study proteins in amorphous and solid phases as there is no upper limit to the protein concentration that can be studied. However, the nature of the scattering can change with increasing concentration due to intermolecular correlations, and thus knowledge of the shape of the proteins can be lost as a result of contributions due to time-averaged spatial ordering of proteins in the sample [11]. Many SAS studies of monoclonal antibodies have been carried out to explore various aspects of antibody function, physical chemistry, and manufacturing [12,13,14,15,16,17,18,19,20,21,22,23,24,25,26]. Many of these studies report that single structures exist in solution by modeling the data using heuristic methods that lead to overfitting an underdetermined problem. It is understood that antibodies are flexible molecules in solution [27,28,29] and very few atomic structures of complete antibodies have been determined by X-ray crystallography [22]. The three complete structures of antibodies reported to date consist of two IgG1 structures [30,31] and a single IgG2 structure [32]. These studies considered the antibody structure to be dynamic and the single set of coordinates reported in each case should be considered “snapshots” of likely configurations sampled in solution. Ensembles of structures to model the SAS data of a monoclonal antibody form a more accurate representation of antibody structures in solution [18], and this has been validated by recent studies using atomic force microscopy and individual particle electron tomography [33,34]. SAXS has been used to determine the relative position of domains and characterize the epitope of an antigen–Fab complex [35], but SAS has been under-utilized to characterize antibody–antigen complexes.

Molecular dynamics, Monte Carlo simulation, and physics-based docking protocols to determine protein–protein interactions are viable tools to predict and model experimental data [36]. There are many simulation methods that one can use to enhance sampling and model the physics of the interactions, often using various approximations. Yet it remains a daunting challenge to accurately predict protein–protein interactions for systems of increasing size, and this is complicated further for flexible or disordered molecules. The sampling of antibody structures in solution can be a intractable task using molecular dynamics simulation, as antibodies have ∼20,000 atoms and require hundreds of thousands of water atoms to accurately represent a model system to natively explore conformational space. While advances have been made in the simulation community using specialty processors such as Anton 2 [37] and graphical processing units [38,39,40], generally, systems the size of a single antibody are currently near the upper limit of what one might hope to simulate in a reasonable amount of time if one has access to the specialized computational hardware. That said, molecular simulations using all-atom [18,25,41,42,43,44], coarse-grain [45,46], and colloidal models [23,26,47,48,49,50] can provide insights into antibody structure and dynamics to probe basic biological, physical, and manufacturing properties [23,24,25,26,42,51]. The complexity and tractability of the problem becomes more challenging when one considers the interaction of protein antigens with an ensemble of flexible antibody configurations. There are a variety of tools available to predict antibody–antigen interactions, such as Rosetta, Modeller, and Haddock, among others [52,53,54,55,56]. A recent re-assessment of antibody binding site conformation prediction has provided insight into the viability of computational methods [57].

We have used SAXS and molecular modeling to characterize the stoichiometry and structures of anti-streptavidin IgG2 monoclonal antibody (ASA-IgG2) tetrameric streptavidin (tSA) complexes. A series of starting models of varied compositions and binding arrangements were then subjected to molecular simulation to provide ensembles of structures to compare to SAXS profiles. Ensembles were generated using backbone torsion-angle Monte Carlo (TAMC) sampling to provide tens of thousands of unique ASA-IgG2–tSA complexes that allowed for a thorough representation of the physical space that ASA-IgG2 and bound tSA could occupy, thus enabling the evaluation of structural models by comparison of theoretical SAXS profiles to the experimental data. This in turn improves the viability of models derived from SAXS (or SANS) data, that inherently contains few constraints. By enhancing the resolution of the experimental SAXS profiles using size-exclusion chromatography (SEC) in line with SAXS detection, together in combination with molecular modeling, it will be shown that specific models of ASA-IgG2–tSA complexes in solution are consistent with the experimental SAXS data.

## 2. Results

### 2.1. Binding Affinity Measurements

**Figure 1:**
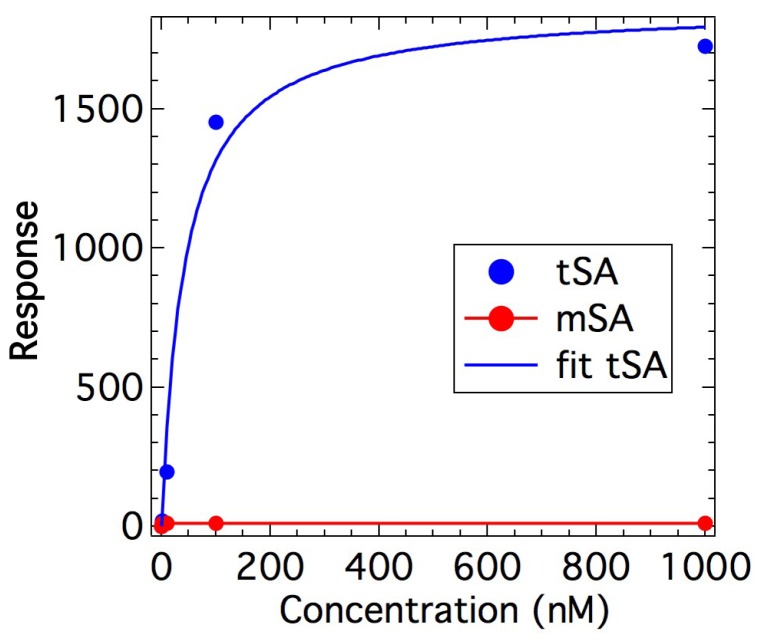
Measured responses for monomeric streptavidin (mSA) and tetrameric streptavidin (tSA) with immobilized anti-streptavidin IgG2 monoclonal antibody (ASA-IgG2).

To assess the specificity of ASA-IgG2 to monomeric streptavidin (mSA) versus tSA, a series of binding affinity measurements were performed using surface-plasmon resonance (SPR) with a Biacore 3000 instrument. Figure 1 shows the measured response after covalently bonding ASA-IgG2 to the surface of the sensor chip and flowing solutions of either mSA and tSA at various concentrations over the chip. The experiment clearly reveals that ASA-IgG2 has no affinity for mSA up to a concentration of 1 µM (M = mol/L). On the contrary, changes in the response were detected after flowing tSA over the immobilized ASA-IgG2. Using a simple 1:1 interaction model, a $K_{D}$ estimate of 40 nM was obtained for tSA and ASA-IgG2.

**Figure 2:**
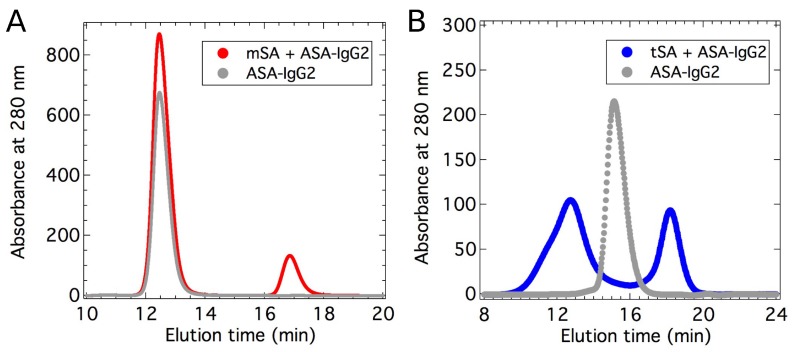
Fast protein liquid chromatography (FPLC) chromatograms of samples with ASA-IgG2 and streptavidin. (**A**) mSA and ASA-IgG2 with a molar ratio of 4:1 (flow rate 0.75 mL/min). (**B**) tSA and ASA-IgG2 with a molar ratio of 5:2 (flow rate 0.50 mL/min). Different concentrations of the free species were used in each chromatogram.

In addition, fast protein liquid chromatography (FPLC) measurements were performed on mixtures of ASA-IgG2 with mSA and tSA, respectively. Figure 2 presents FPLC measurements of ASA-IgG2 in buffer compared to a mixture of ASA-IgG2 with each type of streptavidin. Both chromatograms show the results for ASA-IgG2 only and mixtures of ASA-IgG2 with streptavidin. For the ASA-IgG2 samples, a major peak was observed for the monomer, although about 2% of dimer was observed in the chromatograms. For the mixtures of tSA with ASA-IgG2, two major peaks were observed, representing tSA and the complex of tSA and ASA-IgG2. In the case of mSA and ASA-IgG2, no species eluted before ASA-IgG2, and the two major peaks observed correspond to ASA-IgG2 and mSA. Therefore, no complex of mSA with ASA-IgG2 was detected.

Since no binding was observed between mSA and ASA-IgG2, no further studies were performed with mSA. The following results refer to tSA and the complex formed with ASA-IgG2.

**Figure 3:**
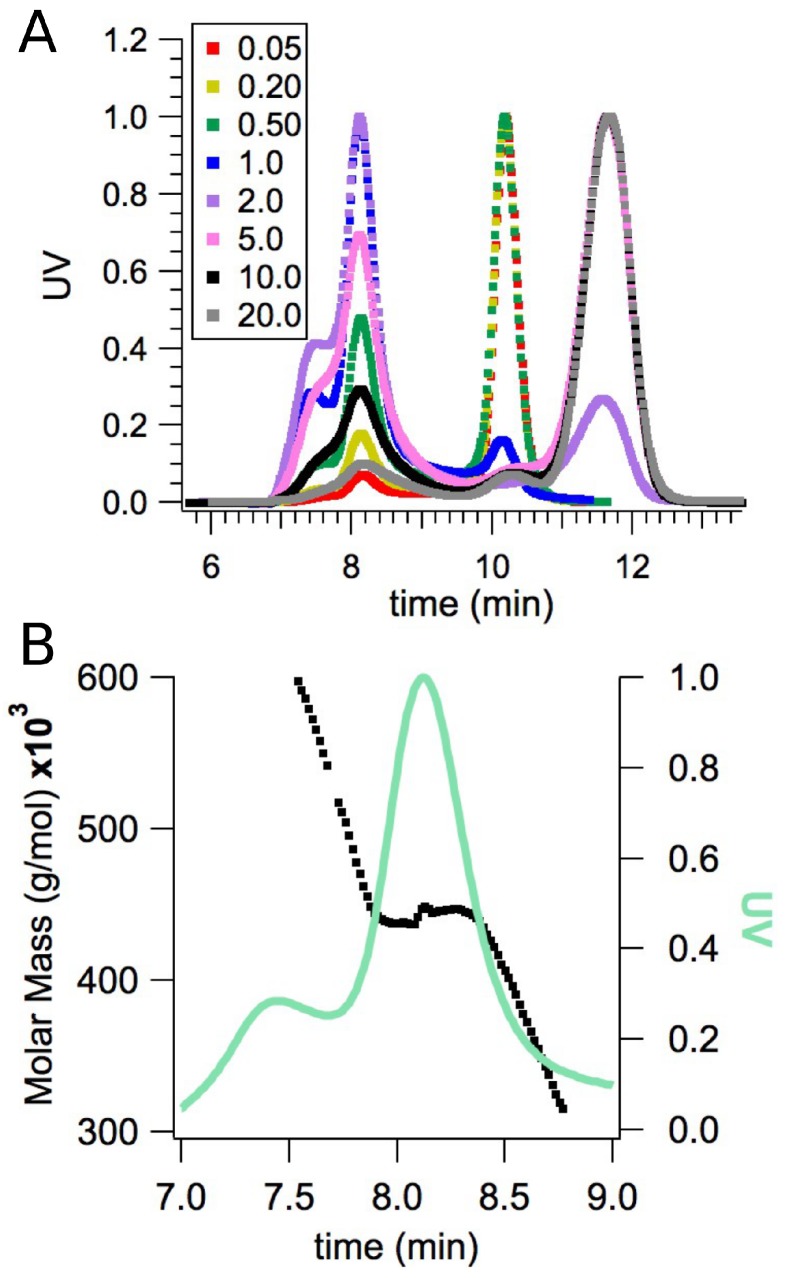
Size-exclusion chromatograms (SECs) of ASA-IgG2 at pH 6.5. (**A**) Different ratios of molar concentration of tSA:ASA-IgG2. (**B**) Molecular weight of the ASA-IgG2–tSA complex peak.

To further characterize the complex of tSA and ASA-IgG2, size-exclusion high-pressure liquid chromatography measurements were coupled with multi-angle light scattering (MALS), known as SEC-MALS, to study various concentration ratios of antigens to antibodies. Figure 3A displays the chromatograms representing the free and bound species after mixing tSA and ASA-IgG2 at different molar ratios. In these chromatograms, the free ASA-IgG2 eluted at about 10 min, whereas tSA eluted after 12 min. The antigen–antibody complex eluted first, which can be seen as a main peak at 8 min with an overlapping left peak or shoulder, depending on the molar ratio. These results suggest that there is not a single complex stoichiometry between tSA and ASA-IgG2. Depending on which free species was in excess and the molar ratios, different amounts of the complex species were formed. When the stoichiometry of the species was close to 1:1, the maximum amount of the complex was formed as most of the ASA-IgG2 and all the tSA were consumed. Regardless, the molecular weight of the main peak in the complex was constant at the center of the peak and during the remainder of the elution, as seen in Figure 3B. Therefore, the most abundant species of the antigen–antibody complex was monodisperse in molecular weight and can be studied by collecting fractions of the main complex peak. Note that the left shoulder in the complex peak became more pronounced with higher molar ratios of tSA:ASA-IgG2 and when free ASA-IgG2 species were depleted.

**Figure 4:**
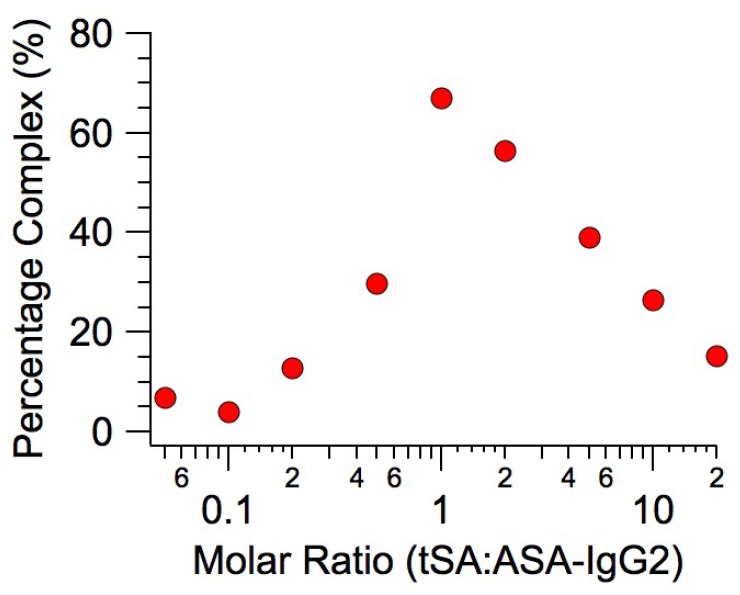
Percentages of the main ASA-IgG2–tSA complex formed at pH 6.5 after mixing ASA-IgG2 and tSA at different molar ratios.

Figure 4 shows the percentage of complex formed after mixing tSA and ASA-IgG2 at different molar ratios. The maximum percentage of the complex was formed when the molar ratio of the species was close to one. Moreover, when the antigen and antibody were mixed at the same molar concentration, very low amounts of the free species were present, suggesting that most of the antigens and antibodies were used to form the complex up to the point that one of the species was depleted. Thus, the equilibrium is favored toward the formation of the complex, in agreement with the low $K_{D}$ estimated from SPR. These results also suggest that molar ratios close to one should be used for the SAXS measurements to obtain higher concentrations of the bound species.

From the SEC-MALS measurements, the molecular weights of the free and bound species were obtained. Table 1 shows the molecular weights of tSA, ASA-IgG2, and the main complex formed. The values for the free species are in agreement with the known values for these molecules. The molecular weight obtained for the main complex is 447 ± 12 kDa. This is an average of eight measurements. The only stoichiometry that results in the experimentally calculated value is a combination of two molecules of tSA and two of ASA-IgG2. Using the values of Table 1, the molecular weight of two molecules of tSA and two of ASA-IgG2 is 447 ± 7 kDa, in agreement with the experimental value for the main complex. For the remainder of the discussion of the results, unless specified, the main complex refers to two ASA-IgG2 molecules associated with two tSA molecules.

**Figure 5:**
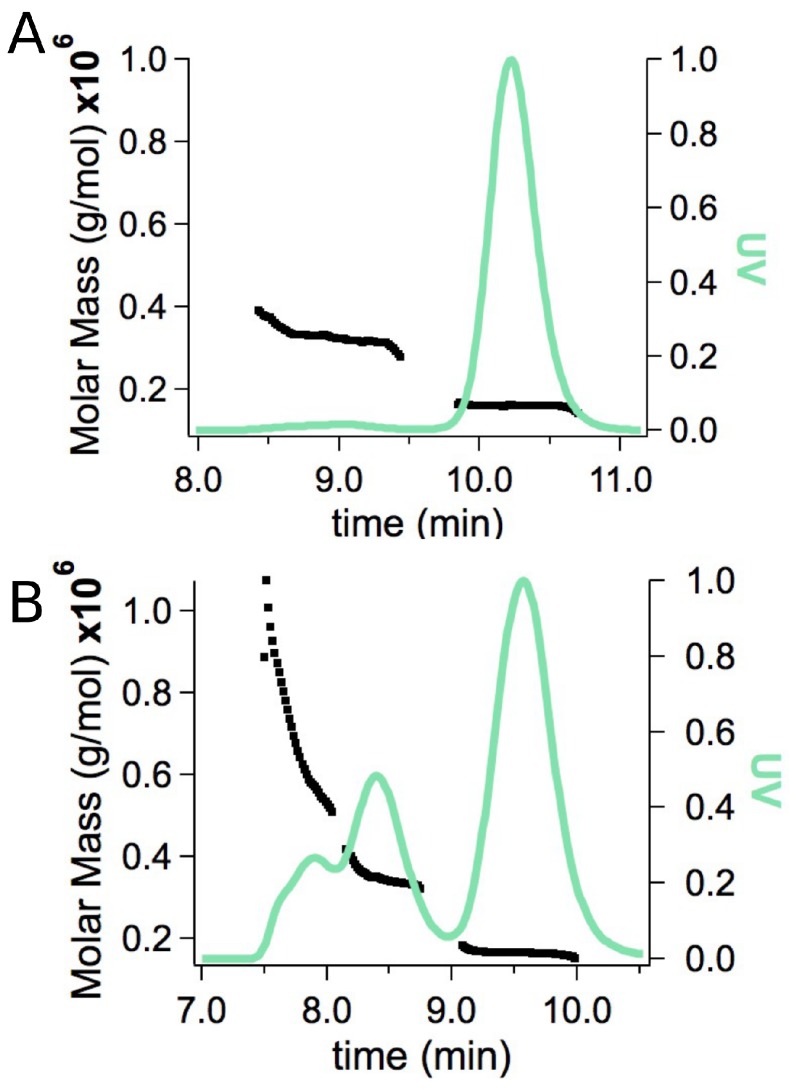
Size-exclusion chromatography coupled with multi-angle light scattering (SEC-MALS) data of ASA-IgG2 at (**A**) pH 6.5 and (**B**) pH 3.0. Light green represents the UV absorption data. Black marks represent the molecular weight of the eluted species.

The formation of the main complex as well as peak shape and separation in the chromatogram were evaluated in acidic pH. However, ASA-IgG2 was unstable at pH 3. Figure 5 shows the SEC chromatogram combined with the MALS data close to neutral and acidic pH. As shown in Figure 2, ASA-IgG2 was mostly monomeric at pH 6.5, with only 2% aggregates. In the case of pH 3.0, the main peak that eluted at 9.5 min represents the ASA-IgG2 monomer, whereas the first elution peaks account for dimers and aggregates of higher molecular weight. Based on the area of the peaks, only 67% of the sample was monomeric at pH 3.0. The peak that eluted before the monomer corresponds to the dimer, and the first peak, which overlaps with the dimer peak, corresponds to aggregates with a wide range of molecular weights. Although acidic pH is not suitable for this system because of aggregation of ASA-IgG2, mixtures of the antigen and the antibody were studied with SEC. Regardless of aggregation, the main complex did not form at acidic pH (data not shown), as the peak area was directly proportional to the prepared concentration of antibody and antigen and neither were associated in a complex. Note that data shown in Figure 1, Figure 2, Figure 3, Figure 4 and Figure 5 correspond to single measurements and do not represent mean values from multiple measurements.

### 2.2. Small-Angle X-ray Scattering

**Figure 6:**
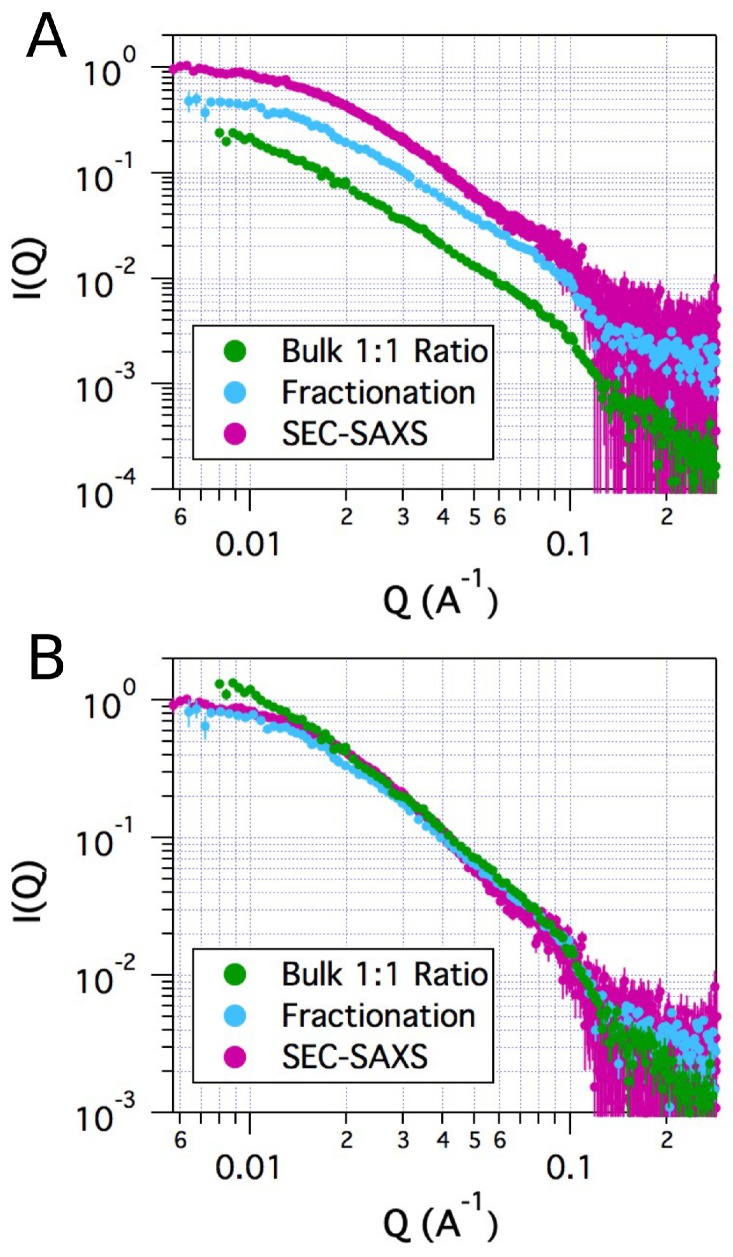
Small-angle X-ray scattering (SAXS) data of the ASA-IgG2–tSA complex at pH 6.5 using different separation methods. (**A**) Profiles are arbitrarily shifted for better visualization. (**B**) Scaled profiles. Error bars correspond to ±1 propagated standard error.

Based on the binding affinity results, SAXS measurements were performed in antigen–antibody mixtures with similar molar ratios at pH 6.5. Figure 6 presents the SAXS data of the antigen–antibody complex using different separation techniques. Although peak fractionation and separation of the main complex peak were clearly needed for SAXS measurements, scattering measurements were performed in bulk (without fractionation), after collecting fractions of the main complex peak (fractionation), and coupling SEC with SAXS (SEC-SAXS). The bulk measurement consisted of a mixture of tSA and ASA-IgG2 at a molar ratio of 1:1, in which the main complex corresponds to 67% of the sample according to the SEC. The SAXS profile for this sample shows a linear slope in the intermediate Q region (0.01–0.1 Å^−1^), which is characteristic of polydisperse systems. In addition, the profile displays an increase in intensity at low Q, indicating the presence of aggregates and large species. The SAXS profile after SEC-fractionation of the main complex peak shows significantly less polydispersity and lower amounts of aggregates. This is confirmed by the SEC-MALS analysis, which shows that 86% of the fractionated sample corresponds to the main complex. Finally, the SEC-SAXS displays the profile of the main complex right after eluting from the SEC column. Although all profiles show similar features, the profile obtained with SEC-SAXS shows the lowest polydispersity and aggregation.

**Figure 7:**
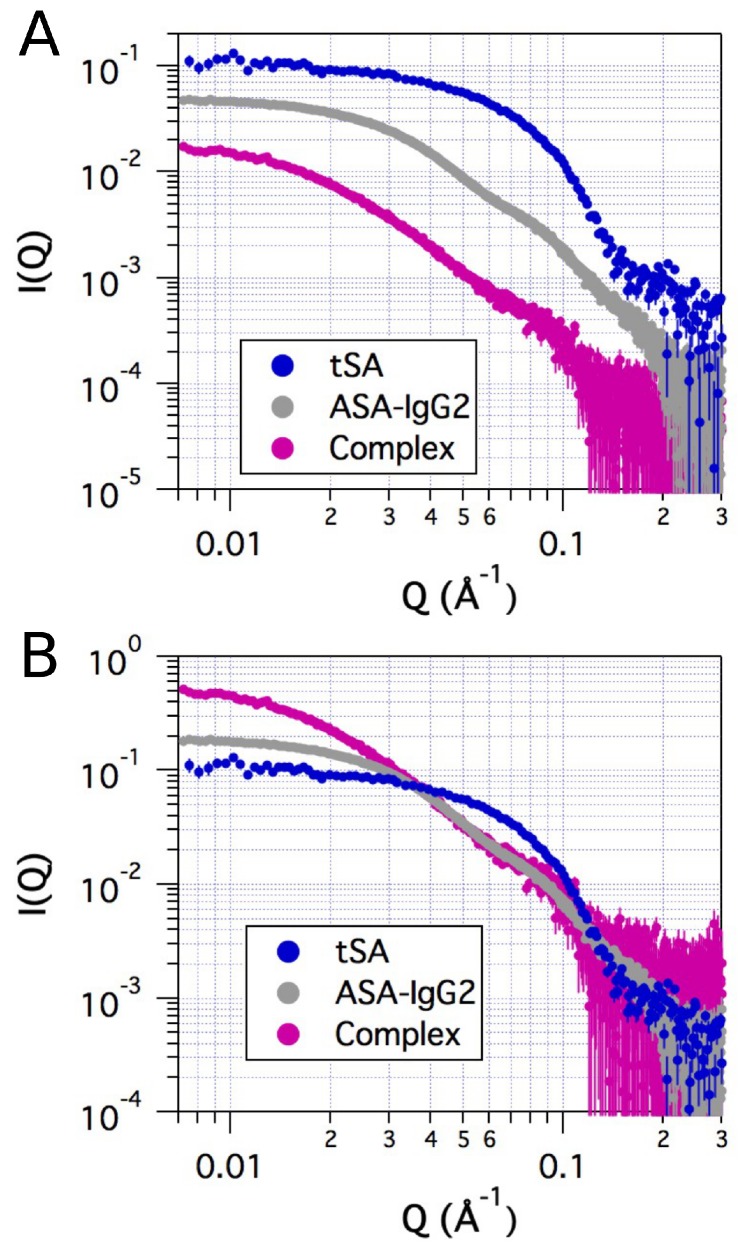
SAXS data of the ASA-IgG2–tSA complex and the free species at pH 6.5. (**A**) Profiles are arbitrarily shifted for better visualization. (**B**) Scaled profiles. Error bars correspond to the ±1 propagated standard error.

Figure 7 shows the SAXS profiles for tSA, ASA-IgG2, and the main complex from the SEC-SAXS measurement. tSA and the ASA-IgG2 had the expected curvature and features in the intermediate Q region for a globular protein and an antibody, respectively [44]. Figure 7B presents the scaled profiles of these species, which show higher intensities at low *Q* for the complex, followed by the ASA-IgG2 and tSA. The low Q intensity is proportional to molecular weight, in agreement with the SEC-MALS results. In addition, the radius of gyration ($R_{g}$) for each sample can be calculated using Guinier analysis. Table 2 displays the results of the Guinier analysis using the SAXS profiles in Figure 6 and Figure 7. As expected, the main complex is the species with the largest size. However, depending on the method used to separate the main complex, some differences are observed. Using fractionation or no separation for the main complex results in larger radii of gyration due to the presence of other higher molecular weight species (see Figure 3). Nonetheless, the method of fractionation provides results that are comparable to those of SEC-SAXS, with a difference in size of 4 Å.

**Figure 8:**
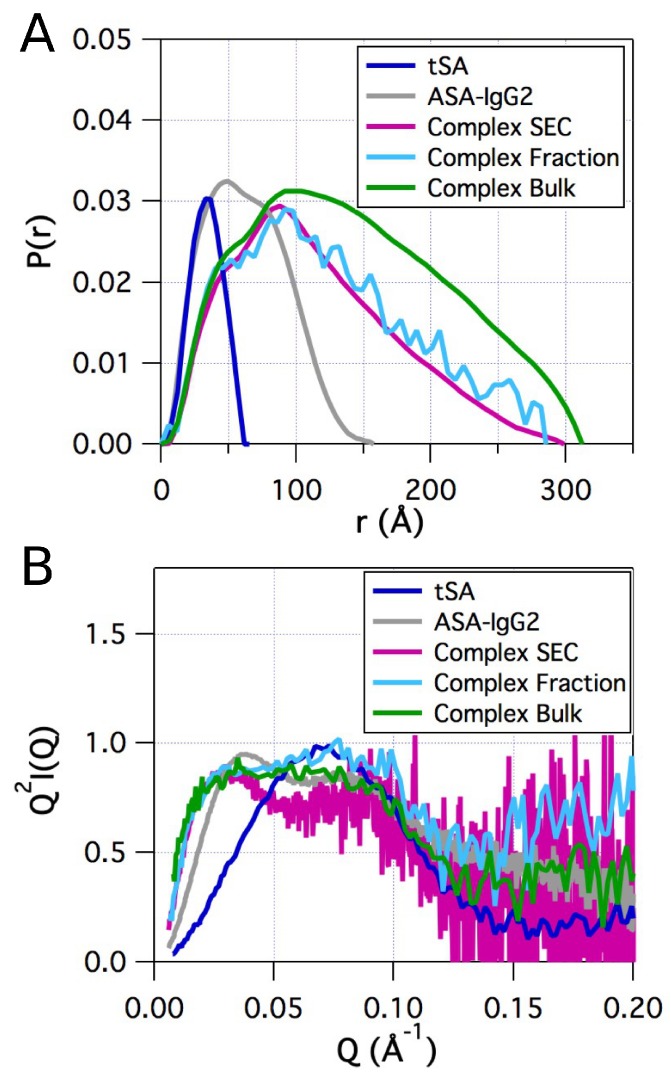
(**A**) Pair distribution function and (**B**) Kratky plot for the ASA-IgG2–tSA complex and its components at pH 6.5.

Figure 8 shows the pair distribution function and Kratky plot for tSA, ASA-IgG2, and the main complex using different separation methods. tSA and ASA-IgG2 show the expected profiles for a globular protein and an antibody, respectively [44]. For the complex, a shoulder is observed in the pair distribution function at about 50 Å and a maximum at 88 Å. The first shoulder matches the maximum of the pair distribution function for tSA and ASA-IgG2, suggesting that it corresponds to distribution of distances in each of the antibody domains and subdomains of the components. On the contrary, the maximum at 88 Å is shifted by 10 Å to larger distances compared to the ASA-IgG2 distribution, which suggests that the peak maximum corresponds to distances between ASA-IgG2 domains and the antigen-binding fragment (Fab) with tSA. A Kratky plot is useful to qualitatively assess the folded or globular state of proteins. ASA-IgG2 has a Kratky plot that is asymmetric, as shown in Figure 8B, indicating some degree of non-globular shape most likely due to inherent flexibility that has been noted for other flexible monoclonal antibodies [18,44]. However, due to the low signal-to-noise in the samples with ASA-IgG2–tSA complexes, it is difficult to judge the degree of flexibility of the complex compared to antibodies alone or to globular proteins. For tSA, a bell-shaped curve characteristic of globular proteins was obtained.

### 2.3. Model Building

**Figure 9:**
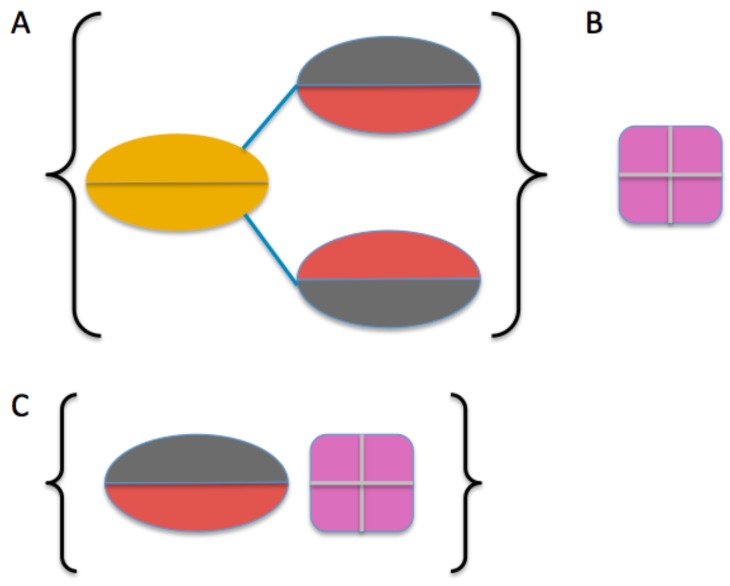

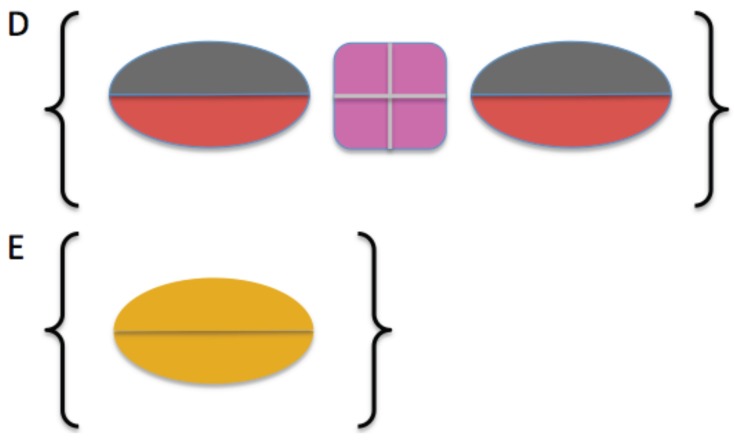
Schematic representation of protein structures used to create models. Fc: orange; Fab light chains: red; Fab heavy chains: grey; linkers between Fc and Fab domains: blue; tSA: mulberry. Brackets indicate that ensembles of structures were created. (**A**) ASA-IgG2; (**B**) tSA; (**C**) Fab–tSA from the docking protocol; (**D**) the Fab–tSA–Fab (Fab2–tSA) complex created by symmetrizing structures from (**C**,**E**) Fc. Coordinates for Fc and Fab were from the original ASA-IgG2 model [18]. Fc: fragment crystallizable region; Fab: antigen-binding region.

**Figure 10:**
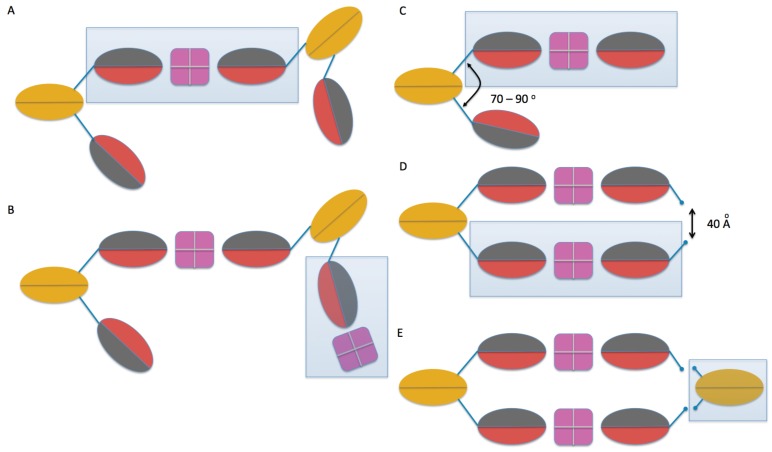
Schematic of model building process for monodentate (**A**,**B**) and bidentate (**C**–**E**) ASA-IgG2–tSA complexes. (**A**) Fc and a secondary Fab structure were aligned to ensembles of Fab2–tSA (depicted in blue box) to create preliminary Fab2–tSA 2ASA-IgG2 structures. (**B**) Ensembles of Fab–tSA were added to structures built in (**A**). Note that the predicted Fab–tSA docked configurations were maintained. (**C**) Fc and secondary Fab structures were aligned to ensembles of Fab2–tSA (depicted in blue boxes) to create preliminary Fab2–tSA single ASA-IgG2 structures. Only structures with Fab–Fc–Fab angles between 70 and 90 degrees were considered. (**D**) Fc and secondary Fab structures were aligned to ensembles of Fab2–tSA (depicted in blue boxes) to create preliminary Fab2–tSA single ASA-IgG2–two Fab structures. Only structures with distances between terminal C-alpha atoms of heavy chain residues 211 of less than 40 Å were considered. (**E**) Fc structures were added to structures from (**D**) that were subsequently energy-minimized and equilibrated using molecular dynamics simulation.

The generation of atomistic models for use to model the SAXS data was carried out in two steps as described in Materials and Methods, and is summarized here. First, a series of building blocks were created in order to systematically create variant models of the various species involved. These structures included ASA-IgG2, tSA, the ASA-IgG2 Fab–tSA complex derived from docking, ASA-IgG2 (Fab)2-tSA derived by symmetrizing the ASA-IgG2 Fab–tSA complex, and the ASA-IgG2 fragment crystallizable region (Fc) domain as shown in Figure 9. As the stoichiometry derived from SEC-SAXS indicates that complexes with two ASA-IgG2 and two tSA molecules are the major components in the main fraction, the building blocks were used to create models of monodentate (single shared tSA) and bidentate (doubly-shared tSA) models, as shown in Figure 10.

### 2.4. Comparison of Models to SAXS Data

**Figure 11:**
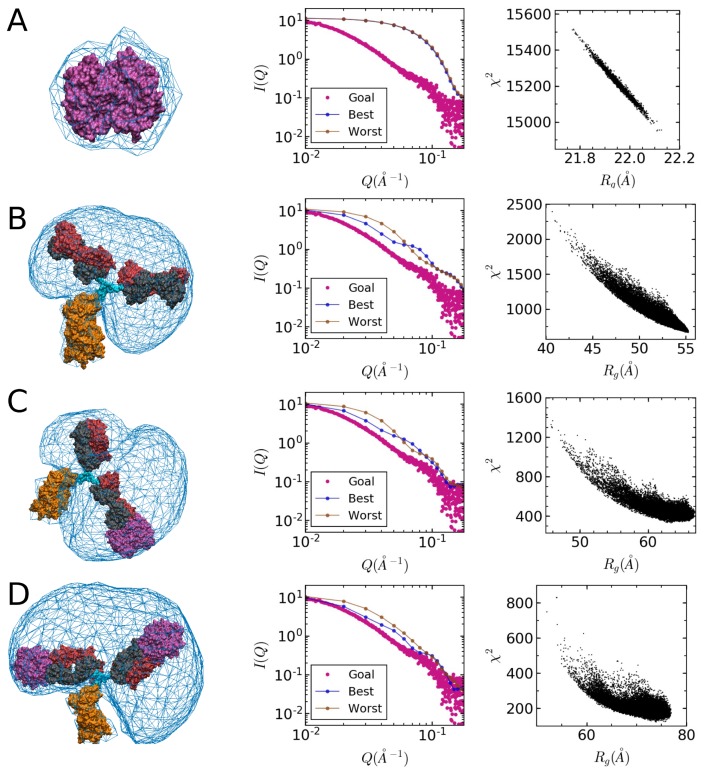

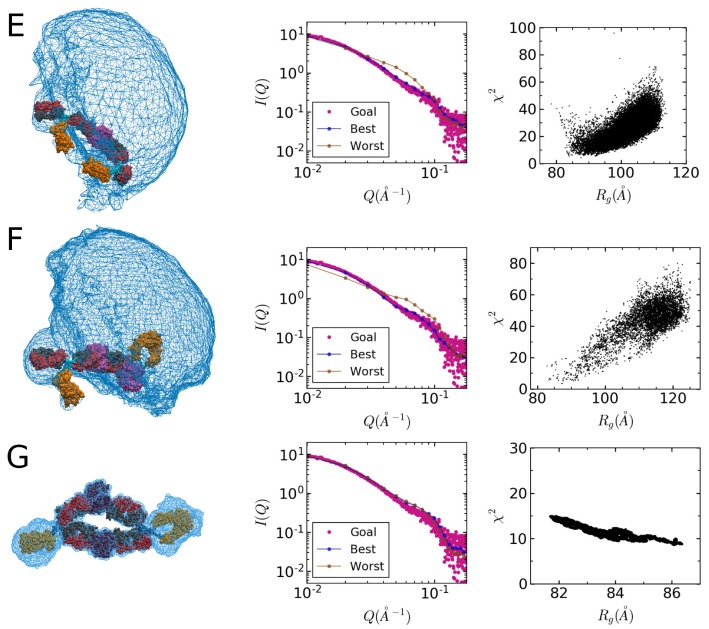
Structures and comparisons of the experimental and simulated scattering profiles. (**A**) tSA; (**B**) ASA-IgG2; (**C**) One ASA-IgG2 and one tSA molecule; (**D**) One ASA-IgG2 and two tSA molecules; (**E**) Two ASA-IgG2 and one tSA molecule; (**F**) Two ASA-IgG2 and two tSA molecules in monodentate configuration; (**G**) Two ASA-IgG2 and two tSA molecules in bidentate configuration. Blue mesh represents the configurational space samples by the ensembles referenced to one Fc. “Goal” represents the experimental data; “Best” represents the best match to the experimental data; and “Worst” represents the worst match to the experimental data. The plots in the right column represent the goodness-of-fit of the models to the experimental data in terms of reduced $\chi^{2}$ as a function of the size of the models via radius of gyration ($R_{g}$).

In order to thoroughly model the SEC-SAXS data, a comprehensive comparison of ensembles of models representing potential molecular species in solution was carried out. While many of these models do not have molecular weights as found in the SEC-MALS data, it is informative to compare the theoretical SAXS profiles to the experimental SEC-SAXS data. As shown in Figure 11 models of tSA, ASA-IgG2, ASA-IgG2–tSA and ASA-IgG2–(tSA)2 are not in agreement with the experimental data as $\chi^{2}$ > 150 for all models for each ensemble, as shown in Figure 11A–D. Note that the blue volumetric densities represent the physical space occupied for each simulation and the single structure depicted within each density plot represents the single best structure from that ensemble determined by comparison of the theoretical SAXS profile to the experimental data. The ensemble of structures of two ASA-IgG2 plus one tSA molecule contained configurations that were by themselves consistent with the SAXS profiles (see Figure 11E) but have to be ruled out based on the measured molecular weight by SEC-MALS.

Evaluation of monodentate (Figure 11F) and bidentate (Figure 11G) models indicates that both contain structures that are in agreement with the SEC-SAXS data. The comparison of the scattering profiles from the structural models to SEC-SAXS profiles indicates that larger extended structures are not consistent with the experimental data and that the most likely set of configurations are compact monodentate or bidentate structures.

## 3. Discussion

We have applied SEC-MALS, and SEC-SAXS with molecular docking and modeling to derive models of the complex formed by a monoclonal antibody and an antigen with the propensity to bind two Fab domains simultaneously. As expected for antibody–antigen complexes, the obtained $K_{D}$ was in the nanomolar range for tSA and ASA-IgG2. The engineered mutations in mSA significantly affected the binding affinity with the ASA-IgG2 and no binding was observed up to micromolar concentrations of the antigen in its monomeric form. Combining SEC with a light scattering detector provided the stoichiometry of the main complex formed by the antibody–antigen complex and the suitable ratios of the free species for the SAXS measurements.

The use of SEC-SAXS improved the homogeneity and monodispersity of the samples, which enabled the evaluation of the structure of the complex using high resolution atomistic models. By using ensembles of viable models obtained from simulation, we were able to explore the specificity of SEC-SAXS to evaluate atomistic models consistent with the solution scattering data. Knowledge of the molecular weight of the species provided discriminating information to rule out models of the two ASA-IgG2 molecules and single tSA molecule in the case where the theoretical SAXS profiles were in agreement with the experimental data.

Finally, we found that compact models of monodentate and bidentate complexes were consistent with the SEC-SAXS data, although further discrimination of whether the monodentate and bidentate complexes are in equilibrium is not available without further experimental constraints. Thus, the use of SEC-SAXS and detailed ensemble modeling is a viable method to characterize antibody–antigen complexes and could have impact for those cases where stoichiometry and/or symmetry allows a discrimination of scattering profiles for putative models. Furthermore, the use of modern docking protocols to create physics-based structures to calculate SAS data in order to compare to SEC-SAXS data is a valuable orthogonal constraint that can be useful in modeling such data.

## 4. Materials and Methods

### 4.1. *Sample Preparation*

ASA-IgG2 was provided by Amgen in frozen solutions of 10 mM sodium acetate buffer, 10 mM acetic acid, 9% *w*/*v* sucrose, with pH 5.2 at concentrations of 30 mg/mL. The tSA (product number S4762 Sigma-Aldrich, St. Louis, MO, USA) was received as a lyophilized powder and stored at −80 °C. The mSA (product number 1385, Kerafast, Boston, MA, USA) is an engineered protein with high affinity to biotin that prevents multivalent interactions. mSA was provided in a 50-mM Tris (pH 7.5) with 150 mM of NaCl buffer and stored at 4 °C for up to two weeks. Frozen samples were thawed overnight at 4 °C one day before usage.

A phosphate buffer solution was prepared using sodium phosphate dibasic anhydrous (product number MSX0720-1, EMD Millipore, Burlington, MA, USA) and potassium phosphate monobasic (product number 231-913-4, Sigma-Aldrich, St. Louis, MO, USA) in Millipore SuperQ water, adjusting the pH to 6.5. Solutions were buffer exchanged using Slide-A-Lyzer dialysis cassettes (product number 66330, Thermo Scientific, Grand Island, NY, USA) with a 3.5-K molecular weight cutoff for mSA. For ASA-IgG2 and tSA samples, solutions were buffer-exchanged using Float-a-Lyzer dialysis devices (product number G235031, SpectrumLabs, Rancho Dominguez, CA, USA) with a 8–10 K molecular weight cutoff. The samples were immersed for at least 10 hours in fresh buffer up to three times while stirring to reach more than 99.9% of the final desired buffer composition. Solutions at pH 3.0 were prepared using a buffer with sodium phosphate dibasic anhydrous (product number MSX0720-1, EMD Millipore, Burlington, MA, USA) and phosphoric acid (product number 79617, Sigma-Aldrich, St. Louis, MO, USA) in Millipore SuperQ adjusted to pH 3.0.

Sample concentration was performed using Amicon Ultra-0.5 centrifugal filters (product number UFC501096, EMD Millipore, Burlington, MA, USA) with a 10-kDa molecular weight cutoff in a swinging bucket centrifuge (product number 75003181, Thermo Scientific, Grand Island, NY, USA) at 4000 relative centrifugal force until reaching the desired concentration. Final protein concentration was measured with a Nanodrop 2000 spectrometer (ND-2000, Thermo Scientific, Grand Island, NY, USA) using percent extinction coefficients ($\varepsilon_{\mathrm{percent}}$) of 24.1, 31.7, and 16.0 for the mSA, tSA, and ASA-IgG2, respectively.

### 4.2. *Binding Measurements*

Binding affinity was assessed using the Biacore 3000. A protein A sensor chip (product number 29127558, GE Healthcare, Pittsburg, PA, USA) was used with a pre-immobilized recombinant protein A that has high affinity for the Fc region of antibodies. A solution of ASA-IgG2 at a concentration of 30 µg/mL was flowed through the cell until reaching a steady-state response, that was used as a baseline. Solutions containing antigen were then injected at concentrations from 1 to 1000 nM for 60 s. Samples and buffer were filtered using a 0.2-µm filter and degassed prior to the measurements. Size exclusion chromatography was performed using a Superdex200 10/300 GL column with an ÄKTA Purifier system (GE Healthcare, Pittsburg, PA, USA). All measurements were performed at 25 °C. This system was used for fractionation, collecting 1 mL of volume(s), and the resulting fractions were concentrated to 2 mg/mL for the SAXS measurements as described above.

High-performance liquid chromatography coupled with multi-angle light scattering was performed using a Thermo Scientific/Dionex U3000. Samples were injected onto a TSKgel G3000SWxl column (product number 08541, Tosoh Bioscience, South San Franciso, CA, USA) and absorbance was measured at 280 nm. After equilibrating the system and the column with the mobile phase, the flow rate was set to 0.8 mL/min for the measurements. Phosphate buffer at pH 6.5 was used as the mobile phase. Light scattering was performed with a DAWN HELEOS II detector (Wyatt Technology, Santa Barbara, CA, USA) at a wavelength of 664 nm. An Optilab T-rEX instrument (Wyatt Technology, Santa Barbara, CA, USA) was used for differential refractive index detection. Data were analyzed with the ASTRA^®^V software (Wyatt Technology, Santa Barbara, CA, USA).

### 4.3. *Small-Angle X-ray Scattering Measurements*

SAXS measurements were performed using an in-house Rigaku X-ray source and the SAXSLab Ganesha platform at the Institute for Bioscience and Biotechnology Research. Samples were loaded into a 96-well plate and sealed with tape to prevent solvent evaporation. Then, 20 µL of each sample was loaded into a 1.3-mm capillary by an automated robot. Sample to detector distance was varied from 0.7 to 1.7 m and a wavelength of 1.5418 Å was used to cover the range 0.005 Å^−1^ < *Q* < 0.45 Å^−1^. Scattered photons were detected with a two-dimensional Pilatus 300 K detector (Dectris, Baden-Dättwil, Switzerland). Data reduction was performed using RAW [58]. This setup was used for all samples, with the exception of those measurements for the complex that were coupled with SEC. Pair distribution functions and Guinier fits were calculated using RAW [58].

SAXS was also performed at BioCAT (beamline 18ID at the Advanced Photon Source, Chicago, IL, USA) with in-line size exclusion chromatography (SEC-SAXS) to separate the species of interest from other species and contaminants, thus ensuring optimal sample quality. Samples were loaded onto a Superdex-200 Increase 10/300 GL column (GE Healthcare, Pittsburg, PA, USA), which was run at 0.75 mL/min, and the eluate, after passing through the UV monitor, was directed through a SAXS flow cell. The SAXS flow cell had a 1.5-mm quartz capillary with 10-µm walls. Scattering intensity was recorded using a Pilatus3 1M detector (Dectris, CH) that was placed ∼3.5 m from the sample resulting in a q-range of 0.0057 to 0.36 Å^−1^. 0.5 s exposures were acquired every 2 s during elution and data was reduced by the beam line specific pipeline that uses the ATSAS program suite [59]. Exposures corresponding to the regions flanking the elution peak were averaged to generate a buffer file which was subtracted from all the exposures. The buffer-subtracted exposures corresponding to the elution peak were used for subsequent analysis. This setup was used for the antigen–antibody complex and the antibody.

The profiles for ASA-IgG2 were consistent between the two SAXS instruments and methods. No radiation damage was observed when using the in-house source or the synchrotron facility. This was confirmed by collecting short exposures as the sample eluted and comparing the radius of gyration and low-Q scattering profile of the initial and final exposures. The free species were measured at concentrations of 1 and 2 mg/mL. No inter-particle interaction effects were observed at the highest concentration used. The complex sample was obtained by mixing the free-species using a molar ratio of 1:1 and separated using SEC-SAXS and fractionation as described in the results.

Certain commercial equipment, instruments, materials, suppliers, or software are identified in this paper to foster understanding. Such identification does not imply recommendation or endorsement by the National Institute of Standards and Technology, nor does it imply that the materials or equipment identified are necessarily the best available for the purpose.

### 4.4. *Molecular Modeling*

A sequence homology model of the ASA-IgG2 with 19,668 atoms, from an earlier study [18], was energy-minimized for 2500 steps, and subject to 1 ns dynamics as described previously. Note that the disulfide bonds in this model correspond to the IgG2-A form [60] characterized by structurally independent Fab domains and hinge region. The resulting structure was used as a starting structure for torsion-angle Monte Carlo (TAMC) studies. The program SASSIE [61] was used to generate 47,319 non-overlapping configurations by sampling backbone angles Φ or Ψ of the amino acid residues 212–214 of the upper hinge region of the heavy chain. Details of the TAMC method applied to ASA-IgG2 are described elsewhere [18].

A model of the tSA protein was created using the coordinates from the crystal structure (PDB ID: 1SWB) [62] obtained from the protein data bank [63] where all missing atoms were added using the program PSFGEN distributed as part of NAMD [64]. The complete model was energy-minimized for 2500 steps with the molecular dynamics program NAMD [64] using the CHARMM22 force field [65]. Subsequently, the structure was immersed in a previously equilibrated 200 Å cubic box of water (using the TIP3P water model [66]) and overlapping waters were removed and a neutralizing number of ions were added. The system was equilibrated at 300 K and 1 bar followed by a production run for 2 ns in the isothermal-isobaric (NPT) ensemble and compared to the SEC-SAXS data shown in Figure 11A.

For clarity, in the remainder of this section, Fab refers to ASA-IgG2 Fab domain and Fc refers to ASA-2 IgG2 Fc domain. Starting with coordinates taken from the TAMC ensemble for the ASA-IgG2 and the final tSA structure from the solvated molecular dynamics simulation, a starting structure for docking was constructed. This structure consisted of a Fab subunit and the tSA protein initially positioned near the Fab paratope region. The Fab subunit consisted of the light chain and residues 1–211 of the heavy chain. The RosettaDock [67] full protocol was used to create 150,000 docking decoys by generating random reorientations of both the Fab and tSA proteins independently. Optimization of side chain conformations of both partners were performed prior to docking using the RosettaDock pre-packing protocol. Subsequent post-analysis involved ranking the decoys using clustering. The set of decoys were sorted by score and the top 10,000 decoys were selected for clustering analysis. The Calibur program [68] was used for clustering with the Rosetta option selected for finding the clustering threshold. The largest cluster was selected, that contained 383 structures.

Assembly of the complexes shown in Figure 9 and Figure 10 involved alignment using the heavy chain of Fab. Structural alignment was carried out using the Align module in SASSIE. For these alignments, care was taken to retain, specifically, the heavy chain which was originally interfaced with tSA from the docking protocol. Moreover, the region of the heavy chains used for alignment was selected to exclude residues which comprised or were near the interfacial region between the tSA and Fab binding site defined by the predictions from docking. Following alignment, atom distances between the pair of aligned molecules were monitored and if any of these distances were less than 8.0 Å, the aligned structure was rejected.

Fab–tSA–Fab complexes (Fab2–tSA) comprising the ensemble shown in Figure 9D were derived by symmetric replication of tSA from the ensemble of Figure 9C. The Fc and secondary Fab were then added to the complex by alignment of the heavy chains of both the full-length ASA-IgG2 structure from the TAMC ensemble (Figure 9A) and the Fab2–streptavidin (SA) complex to give the full-length Fab2–tSA 2 ASA-IgG2 complex shown in Figure 10A. The monodentate ASA-IgG2–tSA complex (Figure 9B) was then constructed by aligning Fab heavy chains of Fab2–tSA structures from the ensemble of Figure 9C and the Fab2–SA 2 ASA-IgG2 complex (Figure 10A). The resulting structures were energy-minimized and subjected to 10 ps Born implicit solvent molecular dynamics (MD). These structures were then used to generate approximately 50,000 accepted configurations in a TAMC simulation. Each TAMC configuration was energy minimized for 2500 steps, and then subjected to 10 ps MD to relax the structural ensemble to compare to SEC-SAXS data shown in Figure 11F.

To construct the bidentate complex, a subset of the TAMC ensemble of full-length ASA-IgG2 with Fab–Fc–Fab angles between 70 and 90 degrees was first collected to provide structures poised to represent more likely candidates for the general bidendate configuration. Subsequently, Fab heavy chains of Fab2–tSA structures from the ensemble of Figure 9C were aligned with heavy chains of both Fab subunits of the selected subset of ASA-IgG2. Only structures with distances between C-alpha atoms of residue 211 of less than 40 Å were retained. Fc structures were then added. The resulting structures were energy-minimized for 2500 steps, and then subjected to 10 ps MD to relax the structural ensemble. A single structure from the ensemble was used for a 10 ns generalized Born MD simulation to provide a trajectory to compare to the SEC-SAXS data shown in Figure 11G.

Theoretical SAS profiles were calculated using the SasCalc [69] module within SASSIE. These profiles were then filtered by calculating reduced $\chi^{2}$ values for each of the SAS profiles relative to a SAS profile calculated for the experimental SAXS data. Nineteen grid points of momentum transfer, Q, between 0 and 0.19 Å^−1^ were used. All the images of protein structures were generated using visual molecular dynamics [70]. 

## Figures and Tables

**Table 1:** Molecular weight of the free and bound species for the main ASA-IgG2–tSA complex

Species	Molecular Weight (kDa)	Number of Measurements
tSA	63.3 ± 0.8	3
ASA-IgG2	160 ± 3	5
main complex	447 ± 12	8

**Table 2:** Guinier Analysis of the SAXS profiles for the free and bound species.

Sample	Method	Radius of Gyration (Å)	$Q_{\min}R_{g}$	$Q_{\max}R_{g}$	$r^{2}$
tSA	Bulk	27.4	0.52	1.3	0.98
ASA-IgG2	SEC	49.2	0.44	1.0	0.99
ASA-IgG2–tSA complex	SEC	84.8	0.56	1.3	0.95
ASA-IgG2–tSA complex	Fractionation	88.9	0.69	1.3	0.91
ASA-IgG2–tSA complex	Bulk	123	1.0	1.2	0.62

## Abbreviations

The following abbreviations are used in this manuscript:

SAXS	small-angle X-ray scattering
SANS	small-angle neutron scattering
SAS	small-angle scattering
SEC	size-exclusion chromatography
SEC-SAXS	size-exclusion chromatography coupled with small-angle X-ray scattering
SEC-MALS	size-exclusion chromatography coupled with multi-angle light scattering
SPR	surface plasmon resonance
MD	molecular dynamics
MC	Monte Carlo
Fab	antigen-binding region
Fc	fragment crystallizable region
IgG	immunoglobulin
SA	streptavidin

## References

[1] Egli M. (2001). Diffraction Techniques in Structural Biology. Current Protocols in Nucleic Acid Chemistry.

[2] Shi Y. (2014). A Glimpse of Structural Biology through X-ray Crystallography. Cell.

[3] Markwick P.R.L., Malliavin T., Nilges M. (2008). Structural Biology by NMR: Structure, Dynamics, and Interactions. PLoS Comput. Biol..

[4] Marion D. (2013). An Introduction to Biological NMR Spectroscopy. Mol. Cell. Proteom..

[5] Stuhrmann H.B., Miller A. (1978). Small-angle scattering of biological structures. J. Appl. Crystallogr..

[6] Svergun D.I., Koch M.H.J. (2003). Small-angle scattering studies of biological macromolecules in solution. Rep. Prog. Phys..

[7] Jacques D.A., Trewhella J. (2010). Small-angle scattering for structural biology—Expanding the frontier while avoiding the pitfalls. Protein Sci..

[8] Stuhrmann H.B. (2008). Small-angle scattering and its interplay with crystallography, contrast variation in SAXS and SANS. Acta Crystallogr. Sect. A.

[9] Whitten A.E., Trewhella J., Foote S.R., Lee W.J. (2009). Small-Angle Scattering and Neutron Contrast Variation for Studying Bio-Molecular Complexes. Micro and Nano Technologies in Bioanalysis: Methods and Protocols.

[10] Krueger S., Chaudhuri B., Muñoz I.G., Urban V., Qian S. (2017). Designing and Performing Biologicial Solution Small-Angle Neutron Scattering Contrast Variation Experiments on Multi-component Assemblies. Biological Small Angle Scattering: Techniques, Strategies and Tips.

[11] Castellanos M.M., McAuley A., Curtis J.E. (2017). Investigating Structure and Dynamics of Proteins in Amorphous Phases Using Neutron Scattering. Comput. Struct. Biotechnol. J..

[12] Perkins S.J., Bonner A. (2008). Structure determinations of human and chimaeric antibodies by solution scattering and constrained molecular modelling. Biochem. Soc. Trans..

[13] Perkins S.J., Okemefuna A.I., Nan R., Li K., Bonner A. (2009). Constrained solution scattering modelling of human antibodies and complement proteins reveals novel biological insights. J. R. Soc. Interface.

[14] Abe Y., Gor J., Bracewell D.G., Perkins S.J., Dalby P.A. (2010). Masking of the Fc region in human IgG4 by constrained X-ray scattering modelling: Implications for antibody function and therapy. Biochem. J..

[15] Ashish, Solanki A.K., Boone C.D., Krueger J.K. (2010). Global structure of HIV-1 neutralizing antibody IgG1 b12 is asymmetric. Biochem. Biophys. Res. Commun..

[16] Mosbæk C.R., Konarev P.V., Svergun D.I., Rischel C., Vestergaard B. (2012). High concentration formulation studies of an IgG2 antibody using small angle X-ray scattering. Pharm. Res..

[17] Lilyestrom W.G., Shire S.J., Scherer T.M. (2012). Influence of the cosolute environment on IgG solution structure analyzed by small-angle X-ray scattering. J. Phys. Chem. B.

[18] Clark N.J., Zhang H., Krueger S., Lee H.J., Ketchem R.R., Kerwin B., Kanapuram S.R., Treuheit M.J., McAuley A., Curtis J.E. (2013). Small-Angle Neutron Scattering Study of a Monoclonal Antibody Using Free-Energy Constraints. J. Phys. Chem. B.

[19] Castellanos M.M., Pathak J.A., Leach W., Bishop S.M., Colby R.H. (2014). Explaining the non-Newtonian Character of Aggregating Monoclonal Antibody Solutions Using Small-Angle Neutron Scattering. Biophys. J..

[20] Tian X., Langkilde A.E., Thorolfsson M., Rasmussen H.B., Vestergaard B. (2017). Small-Angle X-ray Scattering Screening Complements Conventional Biophysical Analysis: Comparative Structural and Biophysical Analysis of Monoclonal Antibodies IgG1, IgG2, and IgG4. J. Pharm. Sci..

[21] Tian X., Vestergaard B., Thorolfsson M., Yang Z., Rasmussen H.B., Langkilde A.E. (2015). In-depth analysis of subclass-specific conformational preferences of IgG antibodies. IUCrJ.

[22] Rayner L.E., Hui G.K., Gor J., Heenan R.K., Dalby P.A., Perkins S.J. (2015). The Solution Structures of Two Human IgG1 Antibodies Show Conformational Stability and Accommodate Their C1q and Fc*γ*R Ligands. J. Biol. Chem..

[23] Yearley E., Zarraga I., Shire S., Scherer T., Gokarn Y., Wagner N., Liu Y. (2013). Small-Angle Neutron Scattering Characterization of Monoclonal Antibody Conformations and Interactions at High Concentrations. Biophys. J..

[24] Yearley E.J., Godfrin P.D., Perevozchikova T., Zhang H., Falus P., Porcar L., Nagao M., Curtis J.E., Gawande P., Taing R. (2014). Observation of Small Cluster Formation in Concentrated Monoclonal Antibody Solutions and Its Implications to Solution Viscosity. Biophys. J..

[25] Hui G.K., Wright D.W., Vennard O.L., Rayner L.E., Pang M., Yeo S.C., Gor J., Molyneux K., Barratt J., Perkins S.J. (2015). The solution structures of native and patient monomeric human IgA1 reveal asymmetric extended structures: Implications for function and IgAN disease. Biochem. J..

[26] Castellanos M.M., Clark N.J., Watson M.C., Krueger S., McAuley A., Curtis J.E. (2016). Role of Molecular Flexibility and Colloidal Descriptions of Proteins in Crowded Environments from Small-Angle Scattering. J. Phys. Chem. B.

[27] Yguerabide J., Epstein H.F., Stryer L. (1970). Segmental flexibility in an antibody molecule. J. Mol. Biol..

[28] McCammon J.A., Karplus M. (1977). Internal motions of antibody molecules. Nature.

[29] Hanson D.C., Yguerabide J., Schumaker V.N. (1981). Segmental flexibility of immunoglobulin G antibody molecules in solution: A new interpretation. Biochemistry.

[30] Harris L.J., Skaletsky E., McPherson A. (1998). Crystallographic structure of an intact IgG1 monoclonal antibody. J. Mol. Biol..

[31] Saphire E.O., Parren P.W.H.I., Barbas C.F., Burton D.R., Wilson I.A. (2001). Crystallization and preliminary structure determination of an intact human immunoglobulin, b12: An antibody that broadly neutralizes primary isolates of HIV-1. Acta Crystallogr. Sect. D.

[32] Harris L.J., Larson S.B., Hasel K.W., Day J., Greenwood A., McPherson A. (1992). The three-dimensional structure of an intact monoclonal antibody for canine lymphoma. Nature.

[33] Chaves R.C., Teulon J.M., Odorico M., Parot P., Chen S.W.W., Pellequer J.L. (2013). Conformational dynamics of individual antibodies using computational docking and AFM. J. Mol. Recognit..

[34] Zhang X., Zhang L., Tong H., Peng B., Rames M.J., Zhang S., Ren G. (2015). 3D Structural Fluctuation of IgG1 Antibody Revealed by Individual Particle Electron Tomography. Sci. Rep..

[35] Wilhelm P., Friguet B., Djavadi-Ohaniance L., Pilz I., Goldberg M.E. (1987). Epitope localization in antigen-monoclonal-antibody complexes by small-angle X-ray scattering. Eur. J. Biochem..

[36] Keskin O., Tuncbag N., Gursoy A. (2016). Predicting Protein–Protein Interactions from the Molecular to the Proteome Level. Chem. Rev..

[37] Shaw D.E., Grossman J.P., Bank J.A., Batson B., Butts J.A., Chao J.C., Deneroff M.M., Dror R.O., Even A., Fenton C.H. Anton 2: Raising the Bar for Performance and Programmability in a Special-Purpose Molecular Dynamics Supercomputer. Proceedings of the SC14: International Conference for High Performance Computing, Networking, Storage and Analysis.

[38] Friedrichs M.S., Eastman P., Vaidyanathan V., Houston M., Legrand S., Beberg A.L., Ensign D.L., Bruns C.M., Pande V.S. (2009). Accelerating Molecular Dynamic Simulation on Graphics Processing Units. J. Comput. Chem..

[39] Salomon-Ferrer R., Götz A.W., Poole D., Le Grand S., Walker R.C. (2013). Routine Microsecond Molecular Dynamics Simulations with AMBER on GPUs. 2. Explicit Solvent Particle Mesh Ewald. J. Chem. Theory Comput..

[40] Stone J.E., Phillips J.C., Freddolino P.L., Hardy D.J., Trabuco L.G., Schulten K. (2007). Accelerating molecular modeling applications with graphics processors. J. Comput. Chem..

[41] Brandt J.P., Patapoff T.W., Aragon S.R. (2010). Construction, {MD} Simulation, and Hydrodynamic Validation of an All-Atom Model of a Monoclonal IgG Antibody. Biophys. J..

[42] Fortunato M.E., Colina C.M. (2014). Effects of Galactosylation in Immunoglobulin G from All-Atom Molecular Dynamics Simulations. J. Phys. Chem. B.

[43] Lapelosa M., Patapoff T.W., Zarraga I.E. (2014). Molecular Simulations of the Pairwise Interaction of Monoclonal Antibodies. J. Phys. Chem. B.

[44] Castellanos M.M., Howell S., Gallagher D.T., Curtis J.E. (2017). Characterization of the NISTmAb Reference Material using Small-Angle Scattering and Molecular Simulation Part I: Dilute Solution Structures. Anal. Bioanal. Chem..

[45] Chaudhri A., Zarraga I.E., Kamerzell T.J., Brandt J.P., Patapoff T.W., Shire S.J., Voth G.A. (2012). Coarse-Grained Modeling of the Self-Association of Therapeutic Monoclonal Antibodies. J. Phys. Chem. B.

[46] Franco-Gonzalez J.F., Ramos J., Cruz V.L., Martinez-Salazar J. (2014). Exploring the dynamics and interaction of a full ErbB2 receptor and Trastuzumab-Fab antibody in a lipid bilayer model using Martini coarse-grained force field. J. Comput.-Aided Mol. Des..

[47] Zhou J., Tsao H.K., Sheng Y.J., Jiang S. (2004). Monte Carlo simulations of antibody adsorption and orientation on charged surfaces. J. Chem. Phys..

[48] Calero-Rubio C., Saluja A., Roberts C.J. (2016). Coarse-Grained Antibody Models for “Weak” Protein–Protein Interactions from Low to High Concentrations. J. Phys. Chem. B.

[49] De Michele C., De Los Rios P., Foffi G., Piazza F. (2016). Simulation and Theory of Antibody Binding to Crowded Antigen-Covered Surfaces. PLoS Comput. Biol..

[50] Corbett D., Hebditch M., Keeling R., Ke P., Ekizoglou S., Sarangapani P., Pathak J., Van Der Walle C.F., Uddin S., Baldock C. (2017). Coarse-Grained Modeling of Antibodies from Small-Angle Scattering Profiles. J. Phys. Chem. B.

[51] Arzenšek D., Kuzman D., Podgornik R. (2015). Hofmeister Effects in Monoclonal Antibody Solution Interactions. J. Phys. Chem. B.

[52] Sivasubramanian A., Sircar A., Chaudhury S., Gray J.J. (2009). Toward high-resolution homology modeling of antibody F(v) regions and application to antibody-antigen docking. Proteins.

[53] Weitzner B.D., Jeliazkov J.R., Lyskov S., Marze N., Kuroda D., Frick R., Adolf-Bryfogle J., Biswas N., Dunbrack R.L., Gray J.J. (2017). Modeling and docking of antibody structures with Rosetta. Nat. Protoc..

[54] Šali A., Blundell T.L. (1993). Comparative Protein Modelling by Satisfaction of Spatial Restraints. J. Mol. Biol..

[55] Dominguez C., Boelens R., Bonvin A.M.J.J. (2003). HADDOCK: A Protein-Protein Docking Approach Based on Biochemical or Biophysical Information. J. Am. Chem. Soc..

[56] Van Zundert G., Rodrigues J., Trellet M., Schmitz C., Kastritis P., Karaca E., Melquiond A., van Dijk M., de Vries S., Bonvin A. (2016). The HADDOCK2.2 Web Server: User-Friendly Integrative Modeling of Biomolecular Complexes. J. Mol. Biol..

[57] Almagro J.C., Teplyakov A., Luo J., Sweet R.W., Kodangattil S., Hernandez-Guzman F., Gilliland G.L. (2014). Second antibody modeling assessment (AMA-II). Proteins Struct. Funct. Bioinform..

[58] Nielsen S.S., Toft K.N., Snakenborg D., Jeppesen M.G., Jacobsen J.K., Vestergaard B., Kutter J.P., Arleth L. (2009). *BioXTAS RAW*, a software program for high-throughput automated small-angle X-ray scattering data reduction and preliminary analysis. J. Appl. Crystallogr..

[59] Petoukhov M.V., Franke D., Shkumatov A.V., Tria G., Kikhney A.G., Gajda M., Gorba C., Mertens H.D.T., Konarev P.V., Svergun D.I. (2012). New developments in the *ATSAS* program package for small-angle scattering data analysis. J. Appl. Crystallogr..

[60] Wypych J., Li M., Guo A., Zhang Z., Martinez T., Allen M.J., Fodor S., Kelner D.N., Flynn G.C., Liu Y.D. (2008). Human IgG2 Antibodies Display Disulfide-mediated Structural Isoforms. J. Biol. Chem..

[61] Curtis J.E., Raghunandan S., Nanda H., Krueger S. (2012). SASSIE: A program to study intrinsically disordered biological molecules and macromolecular ensembles using experimental scattering restraints. Comput. Phys. Commun..

[62] Freitag S., Le Trong I., Klumb L., Stayton P.S., Stenkamp R.E. (1997). Structural studies of the streptavidin binding loop. Protein Sci..

[63] Berman H.M., Westbrook J., Feng Z., Gilliland G., Bhat T.N., Weissig H., Shindyalov I.N., Bourne P.E. (2000). The Protein Data Bank. Nucleic Acids Res..

[64] Phillips J.C., Braun R., Wang W., Gumbart J., Tajkhorshid E., Villa E., Chipot C., Skeel R.D., Kalé L., Schulten K. (2005). Scalable molecular dynamics with NAMD. J. Comput. Chem..

[65] Brooks B.R., Bruccoleri R.E., Olafson B.D., States D.J., Swaminathan S., Karplus M. (1983). CHARMM: A program for macromolecular energy, minimization, and dynamics calculations. J. Comput. Chem..

[66] Jorgensen W.L., Chandrasekhar J., Madura J.D., Impey R.W., Klein M.L. (1983). Comparison of simple potential functions for simulating liquid water. J. Chem. Phys..

[67] Gray J.J., Moughon S., Wang C., Schueler-Furman O., Kuhlman B., Rohl C.A., Baker D. (2003). Protein–Protein Docking with Simultaneous Optimization of Rigid-body Displacement and Side-chain Conformations. J. Mol. Biol..

[68] Li S.C., Ng Y.K. (2010). Calibur: A tool for clustering large numbers of protein decoys. BMC Bioinform..

[69] Watson M.C., Curtis J.E. (2013). Rapid and accurate calculation of small-angle scattering profiles using the golden ratio. J. Appl. Crystallogr..

[70] Humphrey W., Dalke A., Schulten K. (1996). VMD: Visual molecular dynamics. J. Mol. Graph..

